# Genomic Approaches Reveal Pleiotropic Effects in Crossbred Beef Cattle

**DOI:** 10.3389/fgene.2021.627055

**Published:** 2021-03-19

**Authors:** Fernanda M. Rezende, Eduardo Rodriguez, Joel D. Leal-Gutiérrez, Mauricio A. Elzo, Dwain D. Johnson, Chad Carr, Raluca G. Mateescu

**Affiliations:** ^1^Department of Animal Sciences, University of Florida, Gainesville, FL, United States; ^2^Psychiatry Department, University of California, San Diego, La Jolla, CA, United States

**Keywords:** carcass quality, meat quality, WssGBLUP, Angus x Brahman, pleiotropy

## Abstract

Carcass and meat quality are two important attributes for the beef industry because they drive profitability and consumer demand. These traits are of even greater importance in crossbred cattle used in subtropical and tropical regions for their superior adaptability because they tend to underperform compared to their purebred counterparts. Many of these traits are challenging and expensive to measure and unavailable until late in life or after the animal is harvested, hence unrealistic to improve through traditional phenotypic selection, but perfect candidates for genomic selection. Before genomic selection can be implemented in crossbred populations, it is important to explore if pleiotropic effects exist between carcass and meat quality traits. Therefore, the objective of this study was to identify genomic regions with pleiotropic effects on carcass and meat quality traits in a multibreed Angus–Brahman population that included purebred and crossbred animals. Data included phenotypes for 10 carcass and meat quality traits from 2,384 steers, of which 1,038 were genotyped with the GGP Bovine F-250. Single-trait genome-wide association studies were first used to investigate the relevance of direct additive genetic effects on each carcass, sensory and visual meat quality traits. A second analysis for each trait included all other phenotypes as covariates to correct for direct causal effects from identified genomic regions with pure direct effects on the trait under analysis. Five genomic windows on chromosomes BTA5, BTA7, BTA18, and BTA29 explained more than 1% of additive genetic variance of two or more traits. Moreover, three suggestive pleiotropic regions were identified on BTA10 and BTA19. The 317 genes uncovered in pleiotropic regions included anchoring and cytoskeletal proteins, key players in cell growth, muscle development, lipid metabolism and fat deposition, and important factors in muscle proteolysis. A functional analysis of these genes revealed GO terms directly related to carcass quality, meat quality, and tenderness in beef cattle, including calcium-related processes, cell signaling, and modulation of cell–cell adhesion. These results contribute with novel information about the complex genetic architecture and pleiotropic effects of carcass and meat quality traits in crossbred beef cattle.

## Introduction

A common strategy to improve beef production in tropical and subtropical areas is crossbreeding. Approximately 40% of all beef cattle in the United States are raised in the subtropical Southern and Southeastern areas (Cundiff et al., 2012). The combination of high environmental temperature and humidity, greater incidence of parasite-transmitted diseases, and nutritionally lower quality pastures negatively impacts the growth rate and reproductive performance of Taurine (*Bos taurus taurus*) beef cattle breeds (Burrow, 2015). To attenuate these impacts, producers in tropical and subtropical areas use crossbreeding between European Taurine and Zebu (*Bos taurus indicus*) breeds as a strategy to enhance beef production (Lamy et al., 2012). The resulting crossbred animals combine the tropical adaptation of Zebu cattle with the production performance of Taurine cattle, and in tropical and subtropical conditions they frequently perform better than purebred cattle from the parental breeds due to heterosis (Burrow, 2015). In subtropical areas of the United States, Angus × Brahman crosses are preferred for beef production over other Zebu–Taurine combinations (Chase et al., 2004).

Carcass and meat quality (visual and sensory) are two of the most important attributes for the beef industry because they drive profitability and consumer demand. Carcass and meat quality are complex concepts that are described through multiple traits like ribeye area and marbling (carcass quality); tenderness, flavor, and juiciness (visual meat quality); and color, texture, and firmness (sensory meat quality). Each one of these individual component traits are complex in nature, under the control of multiple genes, and influenced by environmental factors. Most of these component traits are challenging and expensive to measure and unavailable until late in life or after the animal was harvested. Genetic improvement of such traits is not viable through traditional phenotypic selection, but these traits are perfect candidates for genomic selection if genetic markers accounting for a large proportion of the additive genetic variation can be identified.

The genetic architecture of carcass quality traits in beef cattle has been more extensively investigated in purebred (Bolormaa et al., 2011; Tizioto et al., 2013; Magalhães et al., 2016; Mateescu et al., 2017) than in crossbred populations (Peters et al., 2012; Lu et al., 2013; Leal-Gutiérrez et al., 2018; Grigoletto et al., 2020). Less information is available on meat quality in both purebred and crossbred populations largely because of the cost and difficulty associated with measuring these traits on a large number of individuals. Genomic selection is being incorporated in an increasingly large number of cattle populations, initially for traits which are routinely recorded to ensure high levels of accuracy. Thus, it is important to explore the existence of pleiotropic effects between these carcass quality and meat quality (visual and sensory) attributes. This will ensure that genomic selection programs targeting carcass quality traits will not negatively affect the meat quality traits. Therefore, the objective of this study was to identify genomic regions with pleiotropic effects on carcass and meat quality traits in a multibreed Angus–Brahman population.

## Materials and Methods

### Cattle Population and Phenotypic Data

The University of Florida Institutional Animal Care and Use Committee approved the research protocol used in this study (number 201003744). The cattle population for this study consisted of 2,384 steers from the University of Florida multibreed Angus × Brahman herd (Elzo et al., 2016) born between 1989 and 2018. The breed composition of animals in this multibreed population ranged from 100% Angus to 100% Brahman, including purebred animals and all crosses in between them.

Steers were transported to a commercial packing plant when they reached 1.27 cm of subcutaneous fat over the ribeye (FOE, cm), where they were harvested under USDA-FSIS inspection. Carcass quality traits available included hot carcass weight (HCW, kg), marbling score, FOE, and rib eye area (REA, cm^2^). Carcasses were ribbed between the 12th and 13th rib and marbling and REA were visually appraised and recorded by graders 48 h postmortem. Marbling (MARB) was graded as follows: Practically Devoid = 100–199, Traces = 200–299, Slight = 300–399, Small = 400–499, Modest = 500–599, Moderate = 600–699, Slightly Abundant = 700–799, Moderately Abundant = 800–899, Abundant = 900–999. Visual meat quality traits recorded included color (COLOR) on a scale of 1 = extremely bright cherry red to 8 = extremely dark, texture (TEXT) on a scale of 1 = very fine to 7 = extremely coarse, and firmness (FIRM) on a scale of 1 = very firm to 7 = extremely soft. All visual meat quality phenotypes were taken by trained personnel between the 12th and 13th ribs, 48 h postmortem and approximately 1 h after ribbing to allow for oxygenation of the *Longissimus* muscle. Given the small number of observations at the high end of the range for COLOR, TEXT, and FIRM, scores 7 and 8 were combined for COLOR, scores 5, 6, and 7 were combined for TEXT, and scores 4 and 5 were combined for FIRM.

One 2.54 cm thick steak from the *longissimus dorsi* between the 12th and 13th ribs was sampled from each animal, and sensory meat quality traits were assessed in a sensory panel according to the American Meat Science Association Sensory Guidelines. Steaks were transported to the University of Florida Meat Science Laboratory where they were aged for 14 days at 4°C and then frozen at -20°C. Prior to sensory panel assessment, steaks were thawed at 4°C for 24 h, and cooked on an open-hearth grill to an internal temperature of 71°C. Sensory panels consisted of 8–11 trained members, and six animals were assessed by each panel. Two 1 cm × 1 cm × 2.54 cm samples from each steak were provided to each panelist. The sensory panel measurements analyzed by the sensory panelists included: tenderness (TEND; 8 = extremely tender, 7 = very tender, 6 = moderately tender, 5 = slightly tender, 4 = slightly tough, 3 = moderately tough, 2 = very tough, 1 = extremely tough), juiciness (JUIC; 8 = extremely juicy, 7 = very juicy, 6 = moderately juicy, 5 = slightly juicy, 4 = slightly dry, 3 = moderately dry, 2 = very dry, 1 = extremely dry), and beef flavor intensity (FLAV; 1 = extremely bland, 2 = very bland, 3 = moderately bland, 4 = slightly bland, 5 = slightly intense, 6 = moderately intense, 7 = very intense, 8 = extremely intense). Average sensory score from all members of the panel for each steak was used as input in the statistical analyses.

A factor analysis was used to identify high percentages of explained common variances between HCW and REA and between FOE and MARB (data not shown). Subsequently, REA and MARB were selected for further analyses based on their economic importance and likelihood of being included as selection objectives in genetic evaluation programs. Using a similar approach, TEND, JUIC, and FLAV were selected to describe the sensory meat quality, and COLOR, TEXT, and FIRM were chosen to explain visual meat quality.

### Genomic Data

DNA was extracted from blood with the QIAamp DNA Blood Mini DNA kit (Qiagen, Hilden, Germany) following the manufacturer’s protocol and stored at −20°C. Genotyping was carried out on 1,038 of the 2,384 animals using the Bovine GGP F250 array (GeneSeek, Inc., Lincoln, NE, United States) which contains 221,115 single nucleotide polymorphisms (SNPs). The SNP markers mapping to the sex chromosomes, with minor allelic frequency (MAF) lower than 0.01% and call rate lower than 90% were excluded. After quality control, 125,042 SNP markers were retained for subsequent genomic analysis.

### Estimation of Additive Genetic Parameters

Average information restricted maximum likelihood (AIREML) variance components, heritabilities, additive genetic correlations, and phenotypic correlations were estimated using single-trait and two-trait single-step genomic best linear unbiased prediction (ssGBLUP) from single-trait and two-trait animal linear mixed models. Computations were performed with the *airemlf90* package from the BLUPF90 family of programs from Ignacy Misztal and collaborators, University of Georgia. The ssGBLUP procedure utilizes all available phenotypic, pedigree and genotypic information (Misztal et al., 2009). Thus, the ssGBLUP mixed model equations require the inverse of the joint pedigree-genomic relationship matrix (**H**^−1^) instead of the inverse of the classical pedigree-based relationship matrix (**A**^−1^). The **H**^−1^ is defined as follows (Legarra et al., 2009; Aguilar et al., 2010):

$${\text{H}}^{-1}={\text{A}}^{-1}+\left[\begin{array}{cc} 0 & 0\\ 0 & {\text{G}}^{-1}-\,{\text{A}}_{22}^{-1} \end{array}\right],$$

where **G**^−1^ is the inverse of the genomic relationship matrix and ${\text{A}}_{22}^{-1}$ is the inverse of the pedigree relationship matrix for genotyped animals. The **G** matrix was constructed based on VanRaden (2008), assuming allelic frequencies from the current population:

$$G\,=\,\frac{\text{ZZ}{}^{\prime }}{2\,\sum p_{i}\,\left(1-p_{i}\right)}$$

where **Z** is a centered incidence matrix of genotype covariates (0,1,2), and 2∑*p*_*i*_(1−*p*_*i*_) is a scaling parameter in which *p*_*i*_ is the frequency of the reference allele at the *i*th SNP. To avoid singularity issues, **G** inverse was built as **G**^−1^ = (0.95**G** + 0.05**A**_22_)^−1^.

The single-trait and two-trait animal mixed models used in this study included the direct additive genetic and residual as random effects, year of birth as a class effect, and age at slaughter as a covariate, except for TEND and FLAV where age at slaughter was not significant. The single-trait animal mixed models were as follows:

y=X⁢b+Z⁢u+e,

where *y* is a vector of phenotypic records, **X** is an incidence matrix linking phenotypic records to fixed effects, *b* is a vector of fixed effects, **Z** is an incidence matrix relating phenotypic records to direct additive genetic effects, *u* is a vector of random animal direct additive genetic effects, and *e* is a vector of random residuals. The random vectors *u* and *e* were distributed as $u\,∼N\,\left(0,\text{H}\,\sigma_{u}^{2}\right)$ and $e\,∼N\,\left(0,\text{I}\,\sigma_{e}^{2}\right)$, where $\sigma_{u}^{2}$ is the direct additive genetic variance, $\sigma_{e}^{2}$ is the residual variance, **H** is the joint pedigree-genomic relationship matrix, and **I** is an identity matrix. Thus, the (co)variance matrix of *u* and *e* random vectors in single-trait models (**V_1_**) was as follows:

$${\text{V}}_{1}=\left[\begin{array}{cc} \text{H}\,\sigma_{u}^{2} & 0\\ 0 & \text{I}\,\sigma_{e}^{2} \end{array}\right]$$

The two-trait animal mixed models used to estimate phenotypic and genetic correlations between pairs of traits included the same fixed and random effects as the single-trait models. However, it was assumed that *u* ∼*MVN*(0,**T**⊗**H**) and *e* ∼*MVN*(0,**R**⊗**I**), where **T** that is the additive genetic (co)variance matrix and **R** that is the residual (co)variance matrix were defined between the two traits under analysis, MVN represents the multivariate normal distribution, and ⊗ denotes the Kronecker product. Thus, the (co)variance matrix of *u* and *e* random vectors was as follows:

$${\text{V}}_{2}=\left[\begin{array}{cc} \text{T}⊗\text{H} & 0\\ 0 & \text{R}⊗\text{I} \end{array}\right]$$

### Genome-Wide Scan for Pleiotropic Effects

Single-trait genome-wide association studies (GWAS) were carried out using the weighted ssGBLUP (WssGBLUP) procedure (Wang et al., 2012) to investigate the relevance of direct additive genetic effects on each of the carcass, sensory, and visual meat quality traits. The WssGBLUP uses an iterative process, which was repeated three times in this study, to estimate SNP effects and weights. In this approach, the weights of SNPs with larger effects increase, while the weights of markers with smaller effects decrease. Briefly, SNP effects and weights for the GWAS were derived as in Wang et al. (2012) as follows:

1. Set the diagonal matrix of SNP variance or weights as identity, **D** = **I**.
2. Construct the **G** matrix: **G** = **ZDZ**′λ, where λ = 1/2∑*p*_*i*_(1−*p*_*i*_).
3. Predict GEBVs using ssGBLUP with *blupf90* package.
4. Convert GEBVs to SNP effects ($\hat{a}$) with *postGSf90* package: a^=⁢k⁢DZ⁢G-1′⁢u^, where $\hat{u}$ is the GEBV of genotyped animals.
5. Compute the weight for each SNP (*d*_*i*_) using a non-linearA variance method: $d_{i}=C\,T^{\frac{\left|{\hat{a}}_{i}\right|}{\sigma \,\left(\hat{a}\right)}-2}$, where CT is a constant for departure from normality equal to 1.05, $\left|{\hat{a}}_{i}\right|$ is the estimated absolute SNP effect, and $\sigma \,\left(\hat{a}\right)$ is the standard deviation of the vector of estimated SNP effects, with the maximum change in SNP variance limited to 10 (VanRaden, 2008; Lourenco et al., 2020).
6. Normalize SNP weights to maintain the additive genetic variance constant.
7. Iterate from step 2, using the obtained weights to compute the **G**-matrix.

Inbreeding was considered in the set-up of **A**^−1^ to avoid using ad-hoc scaling parameters while keeping GEBV within an acceptable level of inflation/deflation (Lourenco et al., 2020). The percentage of the direct additive genetic variance explained by a given SNP window was calculated according to Wang et al. (2012) as:

$$\frac{\text{Var}\,\left(w_{i}\right)}{\sigma_{u}^{2}}\times 100=\frac{\text{Var}\,\left(\sum_{j=1}^{B}Z_{j}\,{\hat{a}}_{j}\right)}{\sigma_{u}^{2}}\times 100$$

where *w*_*i*_ is the additive genetic value of the *i*th1-Mb genomic window, *B* is the total number of adjacent SNPs within the *i*th window, *Z*_*j*_ is the vector of genotypes of the *j*th SNP for all individuals, and ${\hat{a}}_{j}$ is the estimated additive genetic effect for the *j*th SNP within the *i*th window.

The models used to identify genomic windows associated with the carcass, sensory and visual meat quality traits included all fixed and random effects from the variance component models. In addition, these models included phenotypes for all traits other than the target trait as covariates to correct for causality (Li et al., 2006; Leal-Gutiérrez et al., 2018). Genomic windows explaining more than 1% of direct additive genetic variance were considered to be associated with the analyzed trait. Common genomic regions involving overlapping windows associated with two or more phenotypes were considered as pleiotropic regions. Additionally, common genomic regions including overlapping windows explaining more than 1% of the direct additive genetic variance for one trait and between 0.9 and 1% of the direct additive genetic variance for another trait were considered as suggestive pleiotropic regions. In both cases, the direct effect of a genomic region on two or more traits persists even after each trait was adjusted for all remaining traits.

### Functional Analysis

Genes within pleiotropic regions were identified using the Biomart tool from Ensembl genome browser (Zerbino et al., 2018). It was assumed that causative mutations were located within pleiotropic regions detected with the GWAS. Thus, SNP markers with the largest absolute estimated effect across two or more traits within each pleiotropic region were used to identify genes with a pleiotropic effect. A SNP marker was assigned to a particular gene if it was located within the gene. Gene ontology (GO) terms for all genes inside the pleiotropic regions were also retrieved from the Ensembl database to help determine biological functions and possible mechanistic pathways influencing carcass and meat quality traits. GO and pathway enrichment and clustering analyses of all annotated genes within pleiotropic regions were carried out using the PANTHER Overrepresentation Test (Mi et al., 2019) and the DAVID v6.8 Functional Annotation Tool (Huang et al., 2009).

## Results and Discussion

### Carcass Quality, Visual and Sensory Meat Quality Traits

Table 1 presents numbers of animals, means, SD, minimum and maximum for carcass quality, sensory meat quality, and visual meat quality traits in the multibreed Angus–Brahman population. Similar values were reported for these traits in Brahman and Brahman-influenced populations (Riley et al., 2003; Smith et al., 2007).

**TABLE 1:** Descriptive statistics for carcass, sensory meat quality, and visual meat quality traits in a multibreed Angus–Brahman population.

Trait^1^	N	Mean	SD	Min	Max
**Carcass quality**					
MARB	2,380	410.44	96.89	150	900
REA, cm^2^	2,345	80.72	10.96	47.74	129.04
**Sensory meat quality**					
TEND	1,173	5.44	0.88	2.40	7.63
JUIC	1,173	5.29	0.69	3.00	7.50
FLAV	1,173	5.60	0.47	3.80	7.00
**Visual meat quality**					
COLOR	1,599	3.34	1.66	1	8
TEXT	1,336	2.79	0.85	1	7
FIRM	1,335	2.31	0.81	1	5

*^1^MARB, marbling score; REA, ribeye area; TEND, tenderness score; JUIC, juiciness score; FLAV, beef flavor score; COLOR, color; TEXT, texture; FIRM, firmness.*

Ribeye area and marbling score are economically important for producers, particularly marbling due to its high impact on carcass value set by packers. The average REA (80.72 ± 10.96) and marbling score (410.44 ± 96.89) were comparable to national beef industry averages (Shackelford et al., 2012; Boykin et al., 2017), and similar to data previously reported for the multibreed Angus–Brahman population (Elzo et al., 2012, 2016; Leal-Gutiérrez et al., 2019). This indicates that marbling scores from Angus x Brahman crossbreds are similar to the national beef industry average and include superior carcasses. Further, this similarity in marbling scores is especially important for the Southern United States because crossbreeding with *B. t. indicus* is commonly used to provide some level of adaptability to hot and humid environmental conditions. However, producer profitability may decrease because crossbred cattle with visible *B. t. indicus* characteristics are penalized and their carcasses are discounted (Riley et al., 2005).

While carcass quality is the primary factor determining the value of a carcass in the beef industry supply chain, consumers evaluate beef products at purchase time based on visual quality and at consumption time based on sensory quality. Both the visual and sensory evaluation of the beef product have an important impact on the decision to make a repeated purchase, which is important for sustained or increased demand (Schroeder et al., 2013). Sensory panel members classified steaks from this population to be on average slightly to moderately tender, slightly to moderately juicy and having slightly to moderately intense beef flavor. About 70% of all steaks were rated tender, 91% juicy, and 73% having intense flavor. Color was on average slightly to moderately dark cherry red and similarly texture was fine to moderately fine, and firmness was firm to moderately firm. Overall, 77% of the steaks were rated as dark cherry red or lighter, 80% fine in texture, and 63% firm.

### Genetic Parameters

Table 2 presents single-trait AIREML estimates of genetic variances ($\sigma_{u}^{2}$), residual variances ($\sigma_{e}^{2}$), and heritabilities (*h*^2^) with standard deviation (SD) for carcass quality, sensory meat quality, and visual meat quality traits in the multibreed Angus–Brahman population. Heritability estimates for MARB, REA and TEND were moderate, ranging from 0.43 to 0.53, and consistent with the average of heritability estimates reported in the literature (reviewed by Mateescu, 2014). The low estimates of h^2^ for the other sensory panel and visual meat quality traits (0.11–0.18) were generally consistent with values reported in the literature (Reverter et al., 2003; Dikeman and Pollak, 2005; King et al., 2010; Mateescu, 2014).

**TABLE 2:** Single-trait AIREML estimates of genetic variances ($\sigma_{u}^{2}$), and residual variances ($\sigma_{e}^{2}$), and heritabilities (*h*^2^) with standard deviation (SD) for marbling, rib eye area, juiciness, flavor, tenderness, color, texture, and firmness in a multibreed Angus–Brahman population.

Trait	$\sigma_{u}^{2}$	$\sigma_{e}^{2}$	*h*^2^± SD
MARB	3176.10	3317.30	0.49 ± 0.05
REA, cm^2^	1.05	0.94	0.53 ± 0.05
TEND	0.28	0.36	0.44 ± 0.07
JUIC	0.05	0.29	0.15 ± 0.06
FLAV	0.02	0.15	0.10 ± 0.06
COLOR	0.10	0.56	0.15 ± 0.05
TEXT	0.06	0.43	0.12 ± 0.05
FIRM	0.08	0.34	0.19 ± 0.06

*MARB, marbling score; REA, ribeye area; TEND, tenderness score; JUIC, juiciness score; FLAV, beef flavor score; COLOR, color; TEXT, texture; FIRM, firmness.*

Two-trait AIREML estimates of direct additive genetic and phenotypic correlations between carcass quality, sensory meat quality and visual meat quality traits are presented in Table 3. Ribeye area had consistently the lowest phenotypic correlations with all other traits (−0.05 to 0.04). Positive moderate phenotypic correlations existed between MARB and TEND (0.32), MARB and JUIC (0.32), TEND and JUIC (0.51), TEND and FLAV (0.43), JUIC and FLAV (0.42), and JUIC and COLOR (0.36). Negative moderate phenotypic correlations were estimated between MARB and FIRM (−0.37) and JUIC and FIRM (−0.33). Examination of direct additive genetic correlations between traits in this study is important to understand the challenges and limitations that could result from the inclusion of any of these traits in selection schemes. High and favorable direct additive genetic correlations existed between MARB and a number of other traits (JUIC, FLAV, and FIRM), between all sensory meat quality traits (TEND, JUIC, and FLAV), and between FLAV and TEXT and FLAV and FIRM. The moderate favorable direct additive genetic correlation of 0.21 observed in the present population between two economically important traits MARB and TEND was lower than other estimates of 0.40 (Reverter et al., 2003) and 0.61 (Wheeler et al., 2010). However, this value (0.21) was comparable to estimates by Riley et al. (2003) for Brahman cattle, reinforcing the long-held belief of a unique fat-tenderness relationship in *B. t. indicus* versus *B. t. taurus* cattle. The direct additive genetic correlations reported here between TEND and other visual meat quality traits are supported by other studies in both tropical and temperate breeds (Reverter et al., 2003). Although the relationship between carcass quality traits (particularly marbling) and meat sensory traits (tenderness, juiciness, and flavor) is a very important one, few direct additive genetic correlations have been published to date. This is primarily due to the difficulty and high cost of measuring sensory quality traits in large populations.

**TABLE 3:** Two-trait AIREML estimates of phenotypic (above diagonal) and direct additive genetic (below diagonal) correlations between carcass quality, sensory meat quality, and visual meat quality traits in a multibreed Angus–Brahman population.

Trait^1^	MARB	REA	TEND	JUIC	FLAV	COLOR	TEXT	FIRM
MARB		0.19	0.32	0.32	0.21	0.03	–0.22	–0.37
REA	–0.03		0.10	–0.03	–0.05	0	–0.01	0.04
TEND	0.21	0		0.51	0.43	0.16	–0.08	–0.17
JUIC	0.66	–0.15	0.64		0.42	0.36	–0.01	–0.33
FLAV	0.99	–0.27	0.99	0.99		0.10	0.05	–0.19
COLOR	–0.19	0.02	0	–0.54	–0.37		0.23	–0.19
TEXT	–0.30	0.24	–0.53	–0.99	–0.99	0.02		0.16
FIRM	–0.38	0.24	–0.16	–0.32	–0.99	–0.22	–0.24	

*^1^MARB, marbling score; REA, ribeye area; TEND, tenderness score; JUIC, juiciness score; FLAV, beef flavor score; COLOR, color; TEXT, texture; FIRM, firmness.*

### Genome-Wide Mapping of Pleiotropic Effects

The proportion of the direct additive genetic variance explained by 1-Mb SNP windows for carcass quality, sensory meat quality, and visual meat quality traits across the entire bovine genome is shown in Supplementary Figure 1. The presence of genomic regions associated with two or more traits in this study could be due to the direct and/or indirect effects of these genomic regions on the traits (Li et al., 2006; Leal-Gutiérrez et al., 2018). Direct additive genetic effects are the result of a single causal variant related to multiple traits, independently of its individual effects on each of them and the dependency or causal relationship between different phenotypes. These direct additive genetic effects are considered true pleiotropic effects (Stearns, 2010; Wagner and Zhang, 2011). On the other hand, complex relationships exist between carcass quality, sensory meat quality, and visual meat quality traits and most of them measure some common attributes of the system. For example, the amount of marbling measured by MARB is highly dependent on the variation captured by REA because fat is deposited as the animal grows, and marbling will subsequently impact the meat quality traits (O’Connor et al., 1997; Smith et al., 2007). Because of these dependencies, a genetic variant associated with one trait will show an association with the other traits even if it does not have a direct effect on these other traits. These are considered indirect effects and are expected to disappear when a trait is corrected for the other phenotypes in the system.

**FIGURE 1:**
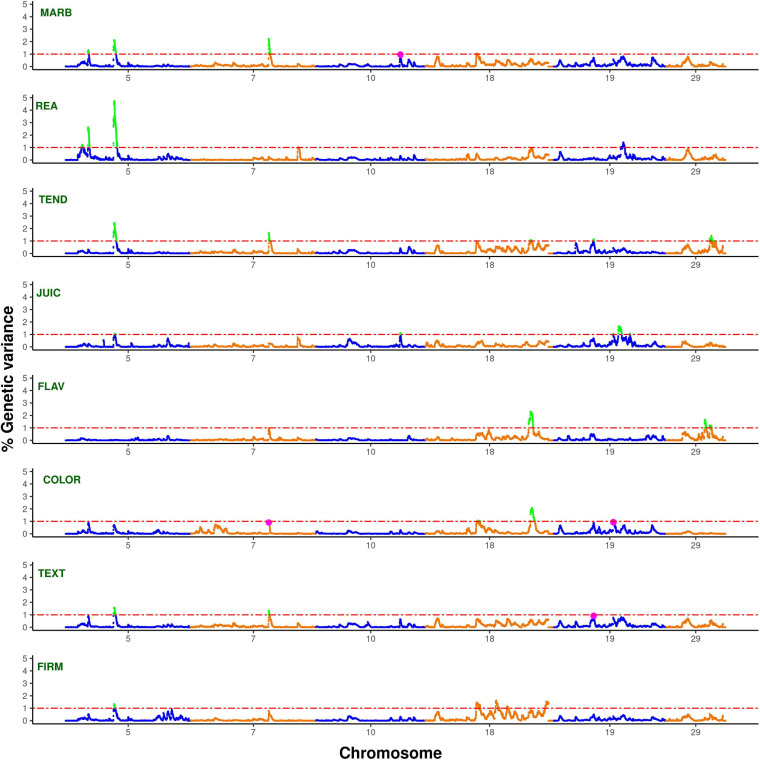
Manhattan plots for bovine chromosomes harboring pleiotropic regions with effect on MARB, REA, TEND, JUIC, FLAV, COLOR, TEXT, and FIRM with significance thresholds indicated at 1% of the additive genetic variance (dash-dotted red line). The variance explained by 1-Mb genomic windows was estimated using single-trait WssGBLUP analyses correcting for indirect effects (i.e., including all remaining traits as covariates). The pleiotropic regions were highlighted in green, and suggestive pleiotropic regions were highlighted in purple.

Conditional genome scan fitting correlated traits as covariates for the trait of interest allows correcting for indirect effects and capturing direct additive genetic effects of genomic regions under analysis (Li et al., 2006). Thus, this approach was implemented to scan for pleiotropic regions affecting carcass quality, sensory meat quality, and visual meat quality traits in the multibreed Angus–Brahman population. The single-trait WssGBLUP analyses correcting for indirect effects (i.e., including all remaining traits as covariates; Figure 1 and Supplementary Figure 2) identified a total of 3,462 non-overlapping 1-Mb genomic windows for MARB, 3,091 for REA, 3,218 for TEND, 3,710 for JUIC, 3,306 for FLAV, 3,381 for COLOR, 3,319 for TEXT, and 3,345 for FIRM. Out of these, 4, 8, 5, 8, 3, 6, 2, and 5 windows explained more than 1% of the direct additive genetic variance for MARB, REA, TEND, JUIC, FLAV, COLOR, TEXT, and FIRM, respectively (Supplementary Tables 1–8). Significant overlapping genomic windows from these analyses with target traits corrected for all other traits are expected to represent genomic regions with pleiotropic effects on the corresponding overlapped traits. Five genomic windows on chromosomes BTA5, BTA7, BTA18, and BTA29 (Table 4) explained more than 1% of the direct additive genetic variance of two or more carcass quality, sensory meat quality, and visual meat quality traits. Moreover, three suggestive pleiotropic regions, defined as regions explaining more than 1% of the direct additive genetic variance for one trait and between 0.9 and 1% for another trait, were identified on BTA10 and BTA19 (Table 4). It is important to point out that these eight pleiotropic regions were previously identified as relevant to carcass quality, sensory meat quality and visual meat quality traits, explaining at least 0.7% of the additive genetic variance of these traits (Supplementary Figure 1).

**TABLE 4:** Genomic windows explaining more than 1% of direct additive genetic variances and pleiotropic genomic regions associated with carcass quality, sensory meat quality, and visual meat quality traits in a multibreed Angus–Brahman population.

BTA	Start location	End location	Trait	Variance Explained, %
**Pleiotropic Window BTA5: 26,723,850-27,497,503**				
5	26,723,850	27,719,719	MARB	1.31
5	26,497,783	27,497,503	REA	2.63
**Pleiotropic Window BTA5: 56,183,908-56,925,298**				
5	55,929,423	56,925,298	MARB	2.12
5	55,947,945	56,939,150	REA	4.72
5	56,081,838	57,079,618	TEND	2.45
5	56,183,908	57,182,379	JUIC	1.06
5	55,929,423	56,925,298	TEXT	1.58
5	55,929,423	56,925,298	FIRM	1.33
**Pleiotropic Window BTA7: 51,559,142-52,520,697**				
7	51,534,263	52,520,697	MARB	2.22
7	51,559,142	52,520,697	TEND	1.65
7	51,364,596	52,357,001	COLOR	*0.92*
7	51,534,263	52,520,697	TEXT	1.34
**Suggestive^1^ Pleiotropic Window BTA10:76,188,006-77,186,559**				
10	76,188,006	77,186,559	MARB	*0.98*
10	76,188,006	77,186,559	JUIC	1.11
**Pleiotropic Window BTA18:61,896,649-62,491,546**				
18	61,559,385	62,559,371	TEND	1.09
18	61,492,103	62,491,546	FLAV	2.31
18	61,896,649	62,896,636	COLOR	2.09
**Suggestive^1^ Pleiotropic Window BTA19:26,984,181-27,979,809**				
19	26,984,181	27,979,809	TEND	1.13
19	26,984,181	27,979,809	TEXT	*0.94*
**Suggestive^1^ Pleiotropic Window BTA19:38,188,955-39,131,233**				
19	38,140,728	39,131,233	JUIC	1.04
19	38,188,955	39,167,086	COLOR	*0.95*
**Pleiotropic Window BTA29:43,148,023-43,405,926**				
29	43,148,023	44,147,635	TEND	1.42
29	42,416,823	43,405,926	FLAV	1.22

*^1^Overlapping windows explaining more than 1% of the direct additive genetic variance for one trait and between 0.9 and 1% for another trait. MARB, marbling score; REA, ribeye area; TEND, tenderness score; JUIC, juiciness score; FLAV, beef flavor score; COLOR, color; TEXT, texture; FIRM, firmness. Genomic windows explaining between 0.9 and 1% of direct additive genetic variances are shown in italic font. The pleiotropic or suggestive pleiotropic windows are defined based on overlapping windows. Columns show the chromosome (BTA), the start and end location of the genomic region, the associated trait, and the direct additive genetic variance explained (%).*

Two pleiotropic windows were identified on BTA5. The first one was located at 26.7–27.5 Mb and explained a high proportion of the direct additive genetic variance in REA (2.63%) and MARB (1.31%). The second one was located at 56.2-56.9 Mb and explained 4.72% of the direct additive genetic variance for REA, 2.45% for TEND, 2.12% for MARB, 1.58% for TEXT, 1.33% for FIRM, and 1.06% for JUIC. The first region around 25–28 Mb on BTA5 was previously reported to be associated with numerous carcass and meat quality traits in beef cattle, specifically MARB and REA (McClure et al., 2010; Baeza et al., 2011). The second window on BTA 5 was found to be associated with MARB and REA (Nalaila et al., 2012; Peters et al., 2012; Saatchi et al., 2014), TEND (Casas and Shackelford, 2000), while a more distant region (68.9–69.1 Mb) was associated with juiciness (Gill et al., 2010).

One genomic window located on BTA7 (51.6–52.5 Mb) had pleiotropic effects on MARB (explaining 2.22% of the direct additive genetic variance), TEND (1.65% of the direct additive genetic variance) and TEXT (1.34% of the direct additive genetic variance), and had a suggestive pleiotropic effect on COLOR (0.92% of the direct additive genetic variance). Previous reports also associated this BTA7 region with MARB (McClure et al., 2010; Mateescu et al., 2017), TEND (Allais et al., 2014), and fat color (Bedhane et al., 2019).

The genomic region between 61.9 and 62.5 Mb on BTA18 accounted for 2.31, 2.09, and 1.09% of the direct additive genetic variance for FLAV, COLOR, and TEND, respectively. Although no specific associations with these traits have been reported, this BTA18 chromosomal region was involved with other carcass traits in cattle (Cole et al., 2011; Höglund et al., 2012; Rolf et al., 2012).

A pleiotropic region located on BTA 29 (43.1–43.4 Mb) simultaneously affected TEND (1.42% of direct additive genetic variance) and FLAV (1.22% of direct additive genetic variance). This is an important region because of its reported association with meat quality, in particular TEND, and because it harbors the μ-calpain gene, a well-established candidate gene due to its role in myofibrillar protein degradation.

A suggestive pleiotropic region on BTA10 (76.2–77.2 Mb) explained 1.11 and 0.98% of the direct additive genetic variance for JUIC and MARB. Lastly, two suggestive pleiotropic regions were detected on BTA19 (27.0–28.0 and 38.2–39.1 Mb). The first region was associated with TEND (1.13% of the direct additive genetic variance) and had a suggestive effect on TEXT (0.94% of direct additive genetic variance), while the second region explained 1.04 and 0.95% of direct additive genetic variance genetic variances for JUIC and COLOR, respectively.

### Genes Within Pleiotropic Regions

The pleiotropic genomic regions described above contained about 317 genes (Supplementary Table 9). However, only candidate genes will be described and discussed here. Genes flagged by the top 20 markers within a specific pleiotropic window (i.e., markers with the largest absolute estimated effect across two or more traits), and with a known function directly or indirectly associated with carcass and meat quality traits were defined as candidate pleiotropic genes.

At least two genes in the first pleiotropic region on BTA5 (26.7–27.5 Mb) are directly involved in muscle physiology and lipid metabolism: *Cysteine Sulfinic Acid Decarboxylase* (*CSAD*) and *Tensin-2* (*TNS2*); hence influencing marbling and ribeye area. The *CSAD* gene is involved in taurine biosynthesis. Taurine, although not used in protein synthesis, is the most abundant free amino acid in mammalian tissues and has multiple functions, including skeletal muscular structure and function (Ito et al., 2008, 2010; De Luca et al., 2015) and lipid metabolism, preventing fat deposition (Murakami, 2015; Wen et al., 2019). Tensin plays a role in skeletal–muscle regeneration (Ishii et al., 2013), and may also cooperate with other actin-binding proteins to modulate actin assembly (Lo et al., 1994).

The second pleiotropic region on BTA5 (56.2–56.9 Mb) harbors three candidate genes involved in lipid metabolism and muscle development, namely *Low-Density Lipoprotein Receptor-Related Protein 1* (*LRP1*), *Myosin 1A* (*MYO1A*), and *Nascent Polypeptide-Associated Complex Alpha Subunit* (*NACA*). The *LRP1* gene plays important roles in many cellular and biological processes, including cell growth and lipid metabolism (Dato and Chiabrando, 2018), and regulates muscle fiber development and myoblast proliferation (Lv et al., 2019). *MYO1A* is a well-known gene related to muscle development, whereas the *NACA* gene is involved in the regulation and differentiation of myoblast cells and myogenic lineages (Berger et al., 2012), and lipid metabolism (Cui et al., 2012). In addition, *MYO1A*, *R3H Domain Containing 2* (*R3HDM2*), *Tachykinin 3* (*TAC3*), and *G Protein-Coupled Receptor 182* (*GPR182*) genes were also reported to be simultaneously associated with carcass and meat quality latent variables in the same multibreed Angus–Brahman population (Leal-Gutiérrez et al., 2018). Lastly, two other genes identified as pleiotropic in this region were the *G Protein-Coupled Receptor 182* (*GPR182*) gene that was differentially expressed in the skeletal muscle of finishing pigs fed a lysine-deficient vs. a lysine-adequate diet (Wang et al., 2016), and the *Retinol Dehydrogenase 16* (*RDH16*) gene that is involved in retinol metabolism and seems to be involved in steatosis in Japanese Black cattle (Ishida et al., 2017). This second BTA5 region is of particular importance because of its pleiotropic effects on most of the traits under investigation. The highlighted candidate genes regulate muscle development, myoblast proliferation, and lipid metabolism. In addition to the obvious effect on MARB and REA, these genes could also affect TEND, TEXT and FIRM given the impact of muscle fiber diameter and density on these traits (Pearson, 1990; Lv et al., 2019).

The pleiotropic window identified on BTA7 contains *Protocadherin Beta 1* (*PCDHB1*), which may directly impact marbling, tenderness, and texture. Protocadherins are cell-adhesion molecules and Refoyo-Martínez et al. (2019) found *PCDHB1* to be under selection in cattle. Cadherins are structural proteins and some of them were associated with marbling, suggesting that they play important roles in cell adhesion and differentiation in several bovine tissues (Lim et al., 2011; Caballero et al., 2014; Martignani et al., 2020). In muscle, cadherins could be involved in processes that lead to less tender and visually coarser meat. Consequently, *PCDHB1* could directly influence marbling, tenderness, and texture.

The pleiotropic window identified on BTA18 contains *Retinol Dehydrogenase 13* (*RDH13*) which could affect color and flavor. Vitamin A, or retinol, gives beef a yellowish hue (Daley et al., 2010). Regulation of retinol in muscle by RDH13 would therefore have a direct effect on color. Elevated levels of vitamin A precursors in the diet were associated with altered fatty acid composition of beef (Daley et al., 2010). Additionally, *RDH13* was associated with fat deposition in beef cattle (Lindholm-Perry et al., 2017). The effect of *RDH13* on beef fatty acid composition could have a direct impact on flavor. Another gene in this window is *Ubiquitin Conjugating Enzyme E2 S (UBE2S)*, a member of the ubiquitin-conjugating enzyme family with important roles in protein metabolism and remodeling of adherens junctions. The role of UBE2S in ubiquitin-mediated proteolysis supports the association with TEND and this is further reinforced by a GWA study in Nellore beef cattle which identified the *UBE2S* gene as related to meat tenderness (Carvalho et al., 2017).

The suggestive pleiotropic region on BTA10 contains the *Spectrin Repeat Containing Nuclear Envelope Protein 2* (*SYNE2*) and *Spectrin Beta, Erythrocytic* (*SPTB*) genes. Both genes encode spectrin proteins that bind actin filaments in the cell to the nuclear membrane stabilizing the cell’s nucleus. *SYNE2* was previously identified in the same multibreed Angus–Brahman population as a candidate gene in a region explaining a large percentage of direct additive genetic variances for carcass quality (Leal-Gutiérrez et al., 2018). It is an obvious candidate gene due to its possible role in proteolysis and cell compartmentalization (Zhang et al., 2007). Changes in the expression of *SPTB* were associated with embryonic lethality in cattle (Oishi et al., 2006).

The first suggestive pleiotropic region on BTA19 (27.0–28.0 Mb) contains four genes that may play important regulatory functions in metabolism and gene expression: *Dynein Axonemal Heavy Chain 2* (*DNAH2*), *Chromodomain Helicase DNA Binding Protein 3* (*CHD3*), *Arachidonate 15-Lipoxygenase Type B* (*ALOX15B*), and *Phosphoribosylformylglycinamidine Synthase* (*PFAS*). The *DNAH2* gene codes for a motor protein found in cilia and flagella that was related to intramuscular fat content and carcass weight in pigs (Hlongwane et al., 2020). The CHD3 protein deacetylates histones for chromatin remodeling and may have an important regulatory function. The *ALOX15B* gene plays a role in cell signaling. This lipoxygenase converts arachidonic acid to 15S-hydroperoxyeicosatetraenoic acid, which is involved in G-protein coupled receptor activation and was associated with obesity in humans (Goossens et al., 2017). Finally, *PFAS* is involved in *de novo* synthesis of purines and mutations in this gene were linked to embryonic lethality in cattle (Michot et al., 2017).

The second suggestive pleiotropic region on BTA19 (38.2–39.1 Mb) contains *Pyridoxamine 5’-Phosphate Oxidase* (*PNPO*), *G Protein-Coupled Receptor 179* (*GPR179*), and *Rho GTPase Activating Protein 23* (*ARHGAP23*). The *PNPO* gene regulates vitamin B6 synthesis and mutations in this gene are known to cause seizures (Ciapaite et al., 2020). The GPR179 binds glutamate and ARHGAP23 is a GTPase involved in signal transduction through transmembrane receptors, thus they may have a regulatory function impacting juiciness and color.

A total of 19 genes were annotated in the pleiotropic region identified on BTA29 (43.1–43.4 Mb) and several of them are structural proteins. Genes coding for anchoring proteins, previously identified as associated with meat quality traits by Leal-Gutiérrez et al. (2018), could contribute to tenderization because they allow the attachment of cytoskeletal proteins, plasma and organelle membranes, and extracellular matrix proteins. However, the most important gene in this region is *CAPN*, an essential factor in postmortem muscle proteolysis. Numerous polymorphisms in the *CAPN*-*CAST* system were identified as associated with meat tenderness in various cattle populations (Leal-Gutiérrez and Mateescu, 2019). While no functional mutation was identified in *CAPN*, this gene remains the main candidate gene for meat quality because of its biological role. Many of the genes in this region have been identified as associated with meat tenderness, but more importantly, have been found to interact with each other, co-localize, and have co-expression relationships (Braz et al., 2019).

### Functional Analysis

**FIGURE 2:**
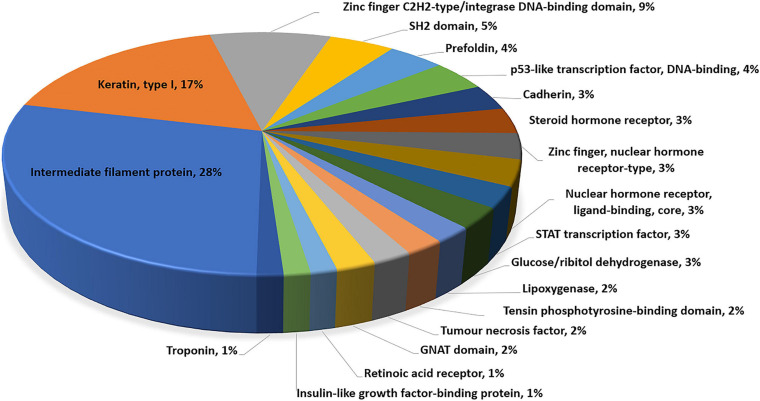
Molecular function analysis of genes located within pleiotropic regions for carcass quality, sensory meat quality, and visual meat quality in a multibreed Angus–Brahman population.

Gene ontology and pathway enrichment analyses were performed to gain insight into the genes located within the most significant pleiotropic regions using PANTHER Overrepresentation Test and the DAVID Functional Classification Clustering tools. The PANTHER classification according to protein family and functionally important domains and sites using the INTERPRO database (Mitchell et al., 2019) is presented in Figure 2. Significant DAVID Functional Annotation Clustering results for the top pleiotropic regions are shown in Table 5. DAVID Functional Annotation Clusters are considered significant above an enrichment score of 1.1.

**TABLE 5:** Top pathways enriched in pleiotropic regions for carcass quality, sensory meat quality, and visual meat quality traits from the DAVID functional annotation module analysis.

Annotation Cluster 1	Enrichment Score: 28.96				
Category	Term	Count	%	*P*-value	FDR
GOTERM_CC_DIRECT	GO:0005882∼intermediate filament	29	10.10	1.35E-36	3.08E-34
GOTERM_CC_DIRECT	GO:0045095∼keratin filament	22	7.67	2.53E-24	2.90E-22
INTERPRO	IPR001664:Intermediate filament protein	41	14.29	5.19E-54	2.41E-51
INTERPRO	IPR018039:Intermediate filament protein, conserved site	33	11.50	7.59E-44	1.76E-41
INTERPRO	IPR002957:Keratin, type I	25	8.71	1.78E-39	2.76E-37
INTERPRO	IPR003054:Type II keratin	15	5.23	6.33E-20	7.36E-18

**Annotation Cluster 2**	**Enrichment Score: 1.97**				
**Category**	**Term**	**Count**	**%**	***P*-value**	**FDR**

GOTERM_BP_DIRECT	GO:0043066∼negative regulation of apoptotic process	8	2.79	0.0815398	1
GOTERM_BP_DIRECT	GO:0008284∼positive regulation of cell proliferation	6	2.09	0.3570181	1
GOTERM_BP_DIRECT	GO:0019221∼cytokine-mediated signaling pathway	3	1.05	0.5191902	1
INTERPRO	IPR000980:SH2 domain	7	2.44	0.0070389	0.142308
INTERPRO	IPR008967:p53-like transcription factor, DNA-binding	6	2.09	4.79E-04	0.018573
INTERPRO	IPR011992:EF-hand-like domain	6	2.09	0.335048	0.978947
INTERPRO	IPR013801:STAT transcription factor, DNA-binding	4	1.39	7.32E-05	0.004865
INTERPRO	IPR001217:STAT transcription factor, core	4	1.39	1.27E-04	0.005355
INTERPRO	IPR013799:STAT transcription factor, protein interaction	4	1.39	1.27E-04	0.005355
INTERPRO	IPR013800:STAT transcription factor, all-alpha	4	1.39	1.27E-04	0.005355
INTERPRO	IPR015988:STAT transcription factor, coiled coil	4	1.39	1.27E-04	0.005355

**Annotation Cluster 3**	**Enrichment Score: 1.65**				
**Category**	**Term**	**Count**	**%**	***P*-value**	**FDR**

GOTERM_BP_DIRECT	GO:0043651∼linoleic acid metabolic process	3	1.05	0.0033093	0.64134
GOTERM_BP_DIRECT	GO:0019372∼lipoxygenase pathway	3	1.05	0.0045868	0.64134
GOTERM_BP_DIRECT	GO:0019369∼arachidonic acid metabolic process	3	1.05	0.0160454	1
GOTERM_MF_DIRECT	GO:0005506∼iron ion binding	3	1.05	0.6926433	1
INTERPRO	IPR020834:Lipoxygenase, conserved site	3	1.05	0.0035124	0.116661
INTERPRO	IPR020833:Lipoxygenase, iron binding site	3	1.05	0.0048667	0.125722
INTERPRO	IPR000907:Lipoxygenase	3	1.05	0.0048667	0.125722

**Annotation Cluster 4**	**Enrichment Score: 1.42**				
**Category**	**Term**	**Count**	**%**	***P*-value**	**FDR**

GOTERM_BP_DIRECT	GO:0007156∼homophilic cell adhesion via plasma membrane adhesion molecules	5	1.74	0.0602773	1
GOTERM_MF_DIRECT	GO:0005509∼calcium ion binding	10	3.48	0.6254933	1
INTERPRO	IPR013164:Cadherin, N-terminal	5	1.74	6.69E-04	0.023919
INTERPRO	IPR020894:Cadherin conserved site	5	1.74	0.0229436	0.426751
INTERPRO	IPR002126:Cadherin	5	1.74	0.0287627	0.477666

**Annotation Cluster 5**	**Enrichment Score: 1.26**				
**Category**	**Term**	**Count**	**%**	***P*-value**	**FDR**

GOTERM_BP_DIRECT	GO:0015031∼protein transport	8	2.79	0.0218376	1
UP_KEYWORDS	Protein transport	9	3.14	0.0682724	0.989189

**Annotation Cluster 6**	**Enrichment Score: 1.19**				
**Category**	**Term**	**Count**	**%**	***P*-value**	**FDR**

GOTERM_MF_DIRECT	GO:0008270∼zinc ion binding	13	4.53	0.8806512	1
GOTERM_MF_DIRECT	GO:0003707∼steroid hormone receptor activity	4	1.39	0.0515058	1
INTERPRO	IPR001723:Steroid hormone receptor	5	1.74	0.005717	0.13292
**Annotation Cluster 7**	**Enrichment Score: 1.16**				
**Category**	**Term**	**Count**	**%**	***P*-value**	**FDR**

UP_KEYWORDS	Protein biosynthesis	5	1.74	0.0726088	0.989189
UP_KEYWORDS	Initiation factor	3	1.05	0.1521805	0.989189
GOTERM_MF_DIRECT	GO:0003743∼translation initiation factor activity	3	1.05	0.2050301	1

**Annotation Cluster 8**	**Enrichment Score: 1.10**				
**Category**	**Term**	**Count**	**%**	***P*-value**	**FDR**

GOTERM_BP_DIRECT	GO:0006351∼transcription, DNA-templated	14	4.88	0.129625	1
GOTERM_BP_DIRECT	GO:0045893∼positive regulation of transcription, DNA-templated	7	2.44	0.1925483	1
GOTERM_MF_DIRECT	GO:0003677∼DNA binding	16	5.57	0.1211263	1
GOTERM_MF_DIRECT	GO:0003700∼transcription factor activity, sequence-specific DNA binding	8	2.79	0.6537571	1

*Statistics associated with GO terms include significance of enrichment or EASE score (*P*-value) and false discovery rate (FDR).*

Overrepresented terms for GO Biological Processes within the most significant pleiotropic regions included “Regulation of Apoptotic Process,” “Regulation of Cell Proliferation,” “Cytokine-Mediated Signaling Pathway,” “Linoleic Acid Metabolic Process,” “Cell Adhesion via Plasma Membrane Adhesion Molecules.” Overrepresented terms for GO Molecular Functions included “Iron Ion Binding,” “Calcium Ion Binding,” “Steroid Hormone Receptor Activity,” “DNA Binding,” “Translation Initiation Factor Activity,” and “Transcription Factor Activity,” Many of these biological pathways were previously reported to be important for carcass quality, meat quality, and tenderness in beef cattle (Guillemin et al., 2012; Mudadu et al., 2016; Ramayo-Caldas et al., 2016; Mateescu et al., 2017; Leal-Gutiérrez et al., 2019). It is important to highlight a few of these enriched pathways given their biological importance in the carcass and meat quality traits under investigation. Numerous genes identified in the significant pleiotropic regions are involved in calcium-related processes such as calcium ion binding, calcium channel, and calcium channel regulator. It was anticipated that calcium and potassium play a major role in meat tenderness because of their contribution to the proteolytic system responsible for muscle contraction and postmortem tenderization. Genes involved in cell signaling and modulation of cell–cell adhesion were also identified as enriched, supporting previous findings in this population (Leal-Gutiérrez et al., 2019). Disruption of structural proteins in the myocytes during and after the aging process is an important determining factor of meat quality. This is via proteolysis of structural proteins such as desmin and talin during aging through the activity of the endogenous μ-calpain-calpastatin system (Koohmaraie and Geesink, 2006; Bee et al., 2007).

## Conclusion

Weighted ssGWAS single-trait genome-wide associations were used to identify genomic regions with pleiotropic effects on carcass quality, sensory meat quality, and visual meat quality traits in a multibreed Angus–Brahman population. Five genomic regions on BTA5, BTA7, BTA18, and BTA29 explained more than 1% of direct additive genetic variance of two or more carcass quality, sensory meat quality, and visual meat quality traits. Moreover, three other suggestive pleiotropic regions were identified on BTA10 and BTA19. A total of 317 genes were identified across all pleiotropic regions. Many of the candidate pleiotropic genes encode anchoring or cytoskeletal proteins, important factors in muscle proteolysis, and key players in cell growth, muscle development, lipid metabolism and fat deposition. A functional analysis of the genes identified in the pleiotropic regions revealed GO terms directly related to carcass quality, meat quality, and tenderness in beef cattle, including calcium-related processes, cell signaling, and modulation of cell–cell adhesion. Results presented here contribute with novel information on the complex architecture of direct additive genetic correlation between carcass and meat quality traits in crossbred beef cattle.

## Data Availability Statement

Publicly available datasets were analyzed in this study. This data can be found here: European Variation Archive website, accession number PRJEB24746.

## Ethics Statement

The animal study was reviewed and approved by University of Florida Institutional Animal Care and Use Committee.

## Author Contributions

FR and RM conceived and designed the study. FR, ER, and RM performed the data analyses and drafted the manuscript. ME edited the manuscript. JL-G, ME, DJ, CC, and RM assisted with data collection. All authors contributed to the article and approved the submitted version.

## Conflict of Interest

The authors declare that the research was conducted in the absence of any commercial or financial relationships that could be construed as a potential conflict of interest.

## Supplementary Material

Supplementary Figure 1 Manhattan plots for MARB, REA, TEND, JUIC, FLAV, COLOR, TEXT and FIRM with significance thresholds indicated at 1% of the additive genetic variance. The variance explained by 1-Mb genomic windows was estimated using a single-trait WssGBLUP analysis.

Supplementary Figure 2 Manhattan plots for MARB, REA, TEND, JUIC, FLAV, COLOR, TEXT and FIRM with significant threshold indicated at 1% of the additive genetic variance. The variance explained by 1-Mb genomic windows was estimated using a single-trait WssGBLUP analysis correcting for indirect effects.

Supplementary Table 1 The number of SNPs and direct additive genetic variance (%) explained in MARB by 1-Mb genomic windows from single-trait WssGBLUP analysis correcting for indirect effects where all other traits were included as covariates.

Supplementary Table 2 The number of SNPs and additive genetic variance (%) explained in REA by 1-Mb genomic windows from single-trait WssGBLUP analysis correcting for indirect effects where all other traits were included as covariates.

Supplementary Table 3 The number of SNPs and additive genetic variance (%) explained in TEND by 1-Mb genomic windows from single-trait WssGBLUP analysis correcting for indirect effects where all other traits were included as covariates.

Supplementary Table 4 The number of SNPs and additive genetic variance (%) explained in JUIC by 1-Mb genomic windows from single-trait WssGBLUP analysis correcting for indirect effects where all other traits were included as covariates.

Supplementary Table 5 The number of SNPs and additive genetic variance (%) explained in FLAV by 1-Mb genomic windows from single-trait WssGBLUP analysis correcting for indirect effects where all other traits were included as covariates.

Supplementary Table 6 The number of SNPs and additive genetic variance (%) explained in COLOR by 1-Mb genomic windows from single-trait WssGBLUP analysis correcting for indirect effects where all other traits were included as covariates.

Supplementary Table 7 The number of SNPs and additive genetic variance (%) explained in TEXT by 1-Mb genomic windows from single-trait WssGBLUP analysis correcting for indirect effects where all other traits were included as covariates.

Supplementary Table 8 The number of SNPs and additive genetic variance (%) explained in FIRM by 1-Mb genomic windows from single-trait WssGBLUP analysis correcting for indirect effects where all other traits were included as covariates.

Supplementary Table 9 Genes located within the pleiotropic regions.
