# Supplementary figures and images for: Genomic Approaches Reveal Pleiotropic Effects in Crossbred Beef Cattle
Source: Front Genet. 2021 Mar 19;12:627055. doi: 10.3389/fgene.2021.627055 (PMC8017557; doi:10.3389/fgene.2021.627055)

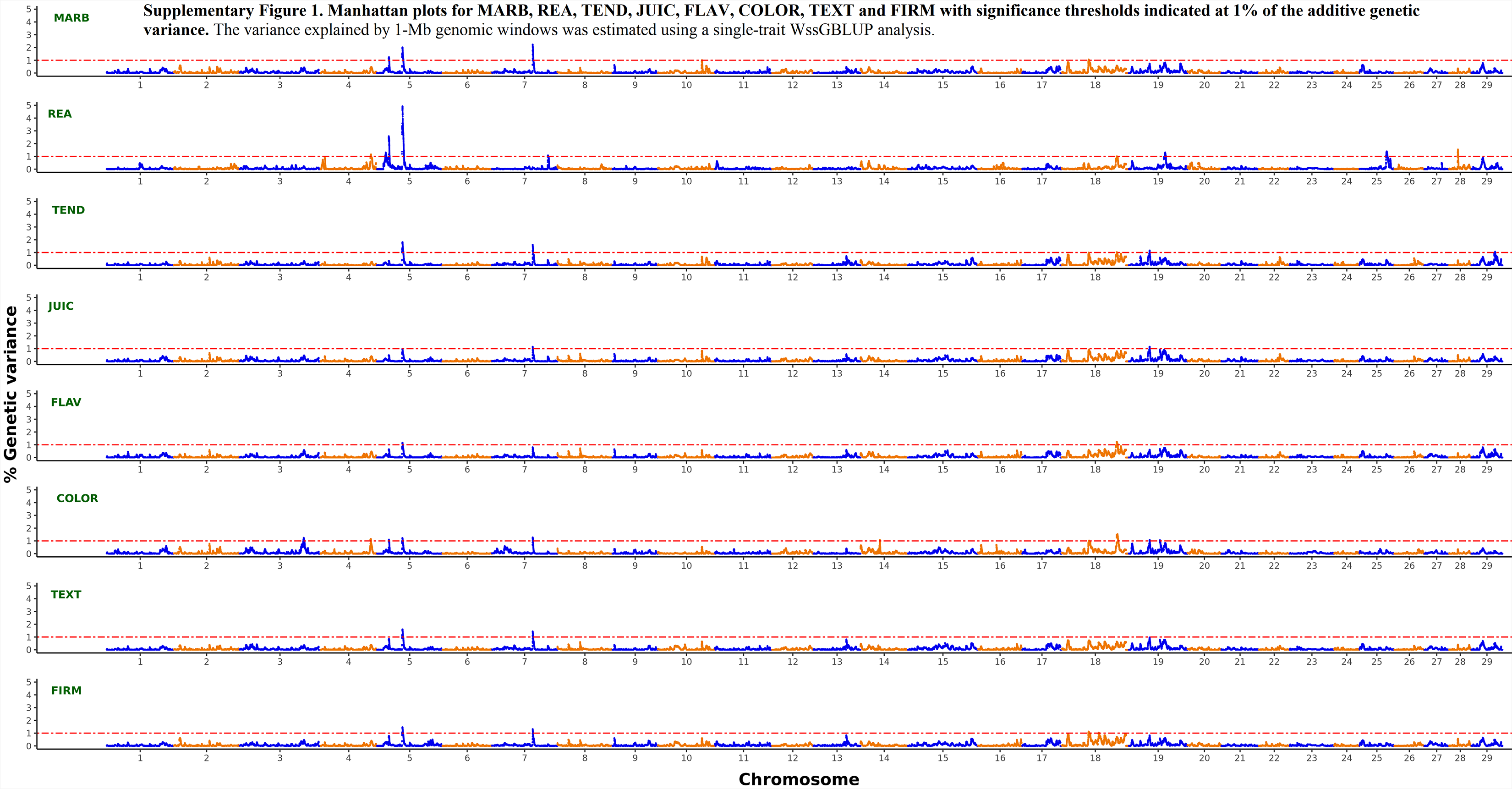

Supplement: Supplementary Figure 1 — Manhattan plots for MARB, REA, TEND, JUIC, FLAV, COLOR, TEXT and FIRM with significance thresholds indicated at 1% of the additive genetic variance. The variance explained by 1-Mb genomic windows was estimated using a single-trait WssGBLUP analysis. [file Image_1.TIF]

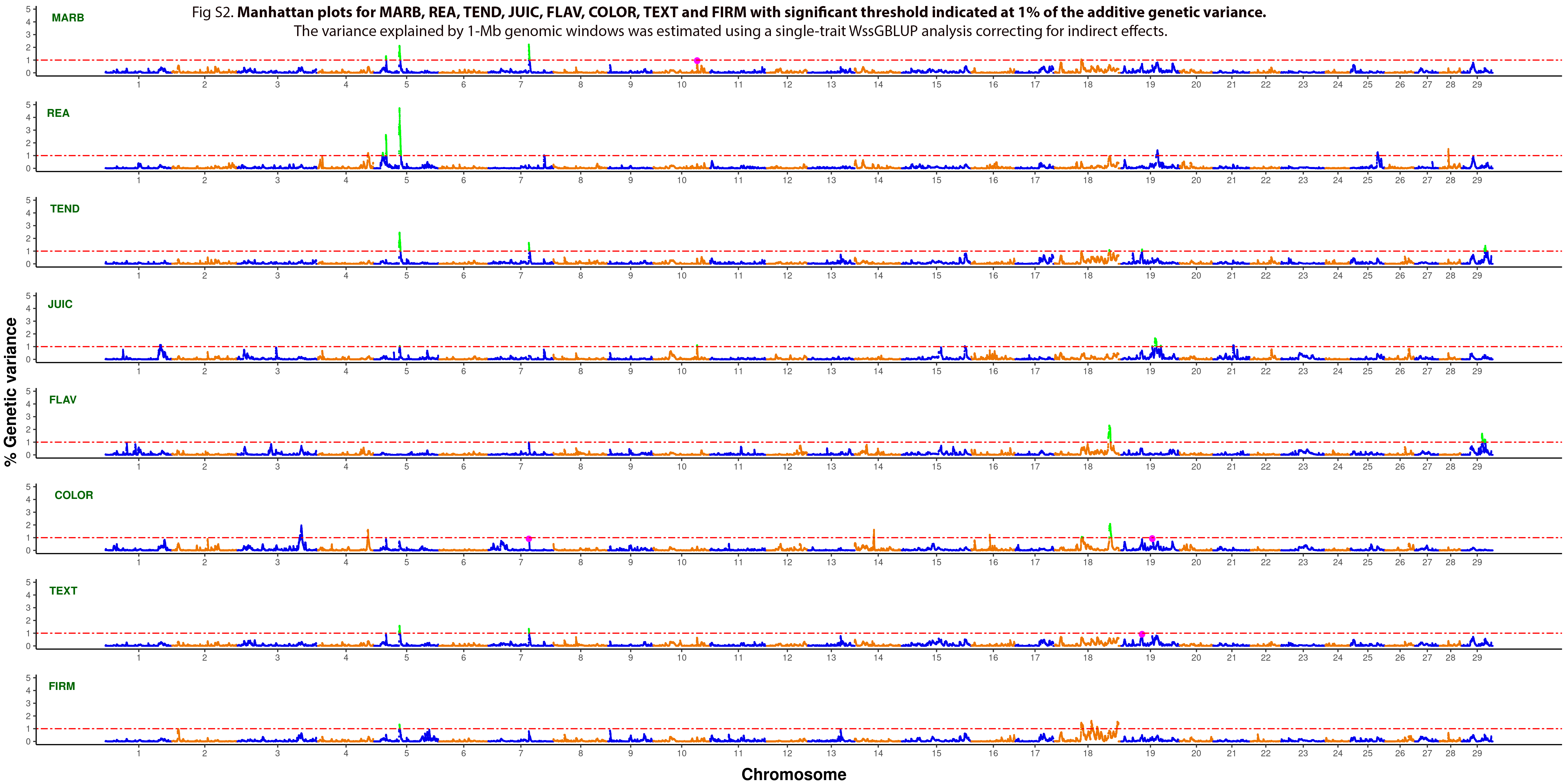

Supplement: Supplementary Figure 2 — Manhattan plots for MARB, REA, TEND, JUIC, FLAV, COLOR, TEXT and FIRM with significant threshold indicated at 1% of the additive genetic variance. The variance explained by 1-Mb genomic windows was estimated using a single-trait WssGBLUP analysis correcting for indirect effects. [file Image_2.png]
